# Lipoprotein Drug Delivery Vehicles for Cancer: Rationale and Reason

**DOI:** 10.3390/ijms20246327

**Published:** 2019-12-15

**Authors:** Jaideep Chaudhary, Joseph Bower, Ian R. Corbin

**Affiliations:** 1 Advanced Imaging Research Center, University of Texas Southwestern Medical Center at Dallas, Dallas, TX 75390, USA; jaideep.chaudhary@utsouthwestern.edu (J.C.);; 2 Department of Radiology, University of Texas Southwestern Medical Center at Dallas, Dallas, TX 75390, USA; 3 Internal Medicine Division of Liver and Digestive Diseases, University of Texas Southwestern Medical Center at Dallas, Dallas, TX 75390, USA

**Keywords:** nanoparticle, lipoprotein, cancer therapy, cancer imaging, cholesterol

## Abstract

Lipoproteins are a family of naturally occurring macromolecular complexes consisting amphiphilic apoproteins, phospholipids, and neutral lipids. The physiological role of mammalian plasma lipoproteins is to transport their apolar cargo (primarily cholesterol and triglyceride) to their respective destinations through a highly organized ligand-receptor recognition system. Current day synthetic nanoparticle delivery systems attempt to accomplish this task; however, many only manage to achieve limited results. In recent years, many research labs have employed the use of lipoprotein or lipoprotein-like carriers to transport imaging agents or drugs to tumors. The purpose of this review is to highlight the pharmacologic, clinical, and molecular evidence for utilizing lipoprotein-based formulations and discuss their scientific rationale. To accomplish this task, evidence of dynamic drug interactions with circulating plasma lipoproteins are presented. This is followed by epidemiologic and molecular data describing the association between cholesterol and cancer.

## 1. Introduction

**Figure 1:**
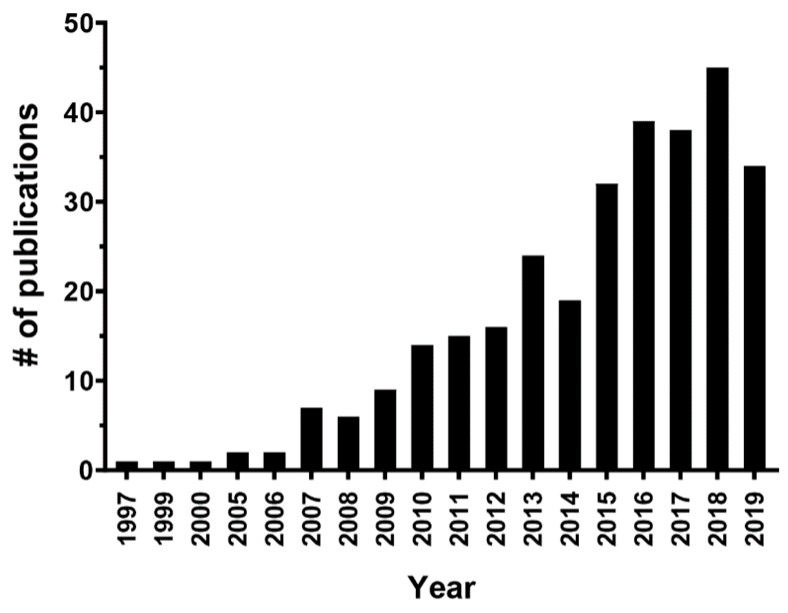
Frequency of publications on lipoproteins nanoparticles and cancer from 1998 to November 2019 in Pubmed using the search term “lipoprotein”, “nanoparticle”, ”cancer”. Exact search criteria: (“lipoproteins” or “lipoprotein”) and (“nanoparticles” or “nanoparticle”) and (“cancer” or “tumor”).

Effective cancer therapy remains a daunting challenge for modern oncology due to the complexities governing tumorigenesis, tumor metastasis, and the limitations associated with current therapies. Over the last three decades colloidal nanocarriers have been implemented in oncology with the promise of providing targeted cancer treatment [1,2,3]. To this end, a diverse array of nanoscale drug-carriers including synthetic (silica, polymers) and natural (lipids, proteins, oligosaccharides) platforms have been designed for cancer drug delivery [4,5]. Among the many investigated nanoparticle systems lipoprotein/lipoprotein-like nanocarriers have become an increasingly attractive and sought-after nanostructures for the delivery of anticancer agents (see Figure 1). Several reviews are available that highlight this unique delivery strategy [6,7,8,9]. Lipoproteins are the main transport system for important lipid molecules such as cholesterol and fatty acids in mammals. The compartmentalized organization of these carriers that enables the transport of native molecules, also makes them amenable for facile incorporation of exogenous compounds [10]. This strategy for cancer drug delivery is not new, in fact back in 1981 Gal et al. proposed that low-density lipoproteins (LDL) could be used as a delivery vehicle for chemotherapeutics and radionucleotides in the management of gynecologic malignancies [11]. Since this citation, numerous researchers from various institutions have utilized lipoprotein-based particles to deliver diverse molecular cargo ranging from contrast media, photodynamic agents, cytotoxic anticancer drugs, small molecule inhibitors, to nucleic acid therapies. Many cell culture and preclinical animal studies have been published demonstrating the feasibility and efficacy of these lipoprotein carriers to transport diagnostic/therapeutic agents to tumors [9,12,13,14,15,16,17]. A few patient studies have even been reported where LDL particles were used to transport Vincristine and Technetium-99 radiolabel in patients with gynecologic and brain malignancies respectively [18,19].

The field of lipoprotein-mediated drug delivery has made numerous advances over the last several years. We have seen the transition from directly using isolated plasma lipoproteins for drug transport to the preferential employment of semi or fully synthetic lipoprotein-based nano vehicles. These nanostructures are typically formulated with natural apoproteins, recombinant apoproteins, synthetic apo-mimetic peptides, commercial lipids, and/or predetermined cargo [20]. This flexibility allows for strict control on ratio and structure of compounds incorporated into the nanoparticle, the size and dimensions of the lipoprotein carrier, thus regulating the physicochemical properties to enable more specific targeting while retaining many of the advantages of the natural lipoproteins. The delivery of nucleic acids with lipoprotein delivery systems (in particular synthetic high density lipoprotein (HDL) carriers) has become an increasingly popular way of performing gene therapy (RNAi, etc.) [14,15,21]. HDL’s natural interaction with SR-B1 provides a means of direct cytosolic delivery of nucleic acid into the target cell. This provides a unique advantage over other particulate delivery system which are significantly hindered by endo-lysosomal trapping. A third and exciting advancement in recent years has been the expanded repertoire of bioactive and contrast agents that can be formulated into the lipoprotein nano-platform. Medical agents ranging from small molecule inhibitors (e.g., Everolimus, Sorafinib) to unique diagnostic nanocrystals of gold, iron oxide, or quantum dots have shown to be efficiently transported by lipoprotein-based vehicles [9,22,23,24]. When this wide array of cargo is combined with the innovations of rerouting lipoproteins to alternate receptor targets, one greatly expands the preview of diagnostic, therapeutic and scientific applications for lipoprotein-mediated drug delivery [25,26].

The rationale behind the tumor-targeting lipoprotein drug delivery approach has often been relegated to oversimplified explanation of increased demand for lipid building blocks needed for membrane synthesis. In many ways this is an incomplete justification for enlisting lipoproteins as drug delivery vehicles. In this review we will highlight several lines of scientific reasoning that support the strategy for lipoprotein mediated drug delivery in oncology. These rationales will include: (i) pharmacological evidence for natural drug-lipoprotein interactions in the mammalian vascular system; (ii) epidemiologic population studies documenting an association between serum cholesterol levels and cancer incidence; (iii) identification of molecular networks that demonstrate the bi-directional signaling between cholesterol and cancer.

## 2. Lipoproteins: Endogenous Lipid Delivery System

**Figure 2:**
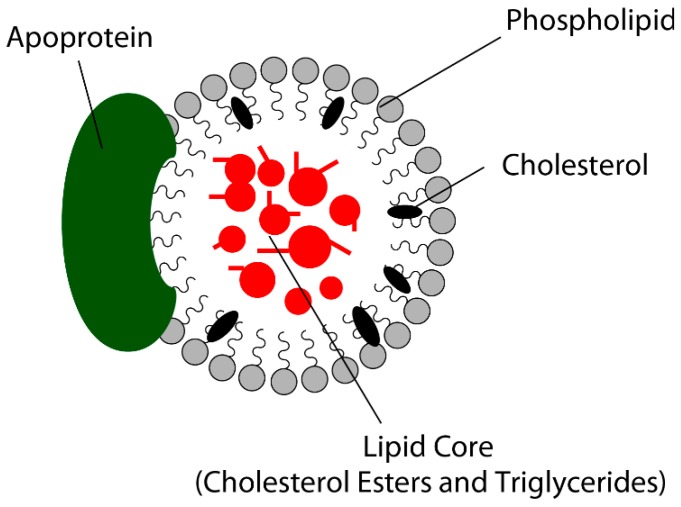
Typical Structure of Lipoproteins. Lipoprotein particles are made up of an apolipoprotein, a phospholipid monolayer with cholesterol particles intercalated in the membrane surrounding a lipophilic core consisting of TGs and cholesterol derivatives.

Plasma lipoproteins are a heterogeneous population of macromolecular aggregates that transport neutral lipids (fat and cholesterol) though the vascular system and extracellular fluid compartments of the body. These spherical lipid-based complexes display a range of physio–chemical properties; however, they have a common structural organization consisting of an apolar core of triglycerides (TG) and cholesterol esters covered by a monolayer of phospholipids and free cholesterol. Interspersed throughout the phospholipid monolayer are specific amphipathic proteins (apolipoproteins) which span the lipid and surrounding aqueous environment (Figure 2). These apolipoproteins provide structural integrity to the framework of the lipoproteins, modulate enzyme activity, as well as serve as ligands for the lipoprotein recognition and cellular uptake [27].

## 3. Classification and Composition of Plasma Lipoproteins

Plasma lipoproteins can be designated into various classes based on numerous physical parameters (e.g., electrophoretic mobility, diameter). The most commonly accepted classification is based on the density of the different lipoprotein species (see Table 1). Accompanying apolipoproteins and their functions are described in Table 2. According to this classification scheme the major density categories include: (i) Chylomicrons (*d* < 0.95 g/mL), these structures are TG-rich emulsion particles (80–88% by weight) containing apolipoprotein B48 that are synthesized by the intestine after a fatty meal. Chylomicrons are the largest particles in the lipoprotein family (80 nm–1 µm in diameter) and have the highest lipid to protein ratio; (ii) Very low density lipoproteins (VLDL, *d* = 0.95–1.006 g/mL), these lipoproteins are also TG rich particles, however, they are synthesized by the liver and contain apolipoprotein B100. They are smaller than chylomicrons (30–80 nm in diameter) and contain relatively less TG but more cholesterol and protein; (iii) Low density lipoproteins (*d* = 1.020–1.063) particles are formed by the intravascular removal of TGs from VLDL (lipoprotein lipase). The LDL core is predominately cholesterol ester molecules. LDL particles are the primary transport mechanism for the delivery of cholesterol to peripheral tissues, and account for 70–80% of circulating cholesterol in humans. Finally, (iv) high density lipoproteins (*d* = 1.063–1.210), these carriers are the smallest (6–12 nm in diameter) member of the lipoprotein family. Their core is mainly composed of cholesterol esters and they are composed of a relatively high proportion of protein (35–56% by weight) consisting primarily of apolipoprotein A1 and A2. The main physiological role of HDL is in the transport of unesterified cholesterol from peripheral tissues back to the liver.

## 4. Drug Interactions with Plasma Lipoproteins

Circulating lipoproteins are highly dynamic macromolecules whose composition and physical structure continually change under the constant flux of interchanging lipids and apolipoproteins. The interchange of lipid and apolipoprotein components between lipoprotein species operates in both fast and slow exchange regimes as passive diffusion and enzyme facilitated transport mediate these processes. During their transit time in the vascular system, other hydrophobic molecules may also associate with the lipoprotein complex. Prime examples of this are the numerous lipophilic vitamins and antioxidants that associate with lipoproteins in the plasma. By far the largest, and probably most important vitamin/antioxidant associated with lipoproteins is α-tocopherol, which averages about 65 molecules per VLDL particle [28], 6 molecules per LDL particle [29], and HDL contains less than one tocopherol per particle [30]. Seminal work by Esterbauer et al. showed that other vitamins/antioxidants were present on LDL only in amounts of about 1/20 to 1/300 of that of α-tocopherol. These vitamins/antioxidants include: γ-tocopherol, β-carotene, α-carotene, lycopene, cryptoxanthin, canthaxanthin, phytofluene, and ubiquinol-10 [29]. These nutrients are also expected to be present at similar low ratios in the other lipoprotein classes.

**Figure 3:**
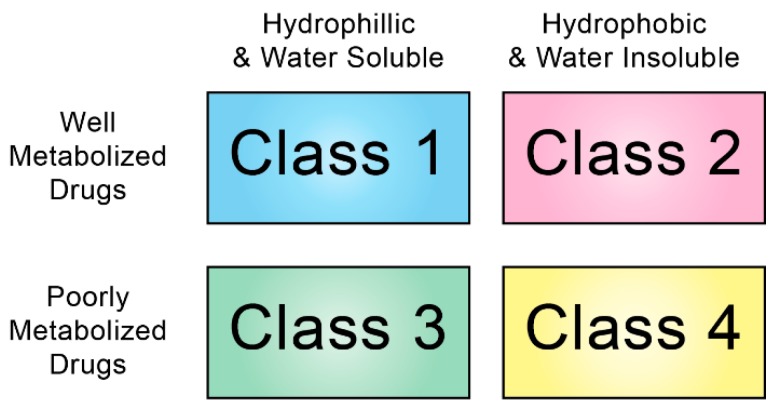
Four Quadrant System for Drug Classification. Biopharmaceutics Drug Disposition Classification System as proposed by Wu and Benet [37].

Hydrophobic/basic drugs are another class of compounds that can bind to lipoproteins in the plasma. Drugs such as cyclosporine A, amiodarone, and amphotericin B are traditionally described in this context [31,32,33]. The biological significance of this association is that the pharmacokinetics, tissue distribution, and pharmacological activity of these drugs can be significantly modified upon binding to plasma lipoproteins [31,34,35,36]. This phenomenon is often overlooked in oncology, but many anticancer drugs do readily associate with circulating lipoproteins. The bulky polycyclic structure of many anticancer drugs enables them to easily cross cellular membranes reach their therapeutic target, but this chemistry also confers poor water solubility. The Biopharmaceutics Drug Disposition Classification System (BDDCS) categorizes drugs based on their water solubility and extent of metabolism (Figure 3) [37]. Class 2 drugs, those having low water solubility and extensive metabolism make up approximately 70% of new drugs in clinical trials and account for 30% of readily-available drugs [38]. In a survey of over 900 BDDCS classified drugs, 265 were categorized as Class 2 drugs and of these 34 (~13%) were identified as anti-cancer agents. Furthermore, studies by Yamamoto et al. indicate that class 2 drugs are more likely to associate with lipoproteins than other classes of drugs [39]. Poorly soluble drugs, like many vitamins/antioxidants, predominantly associate with lipoproteins as a result of thermodynamic pressure. Within the aqueous environment of plasma, poorly soluble compounds will seek other hydrophobic environments in order to minimize their contact with water molecules and maximize the intramolecular van der Waals interactions. Without such apolar environments for escape, poorly soluble compounds will self-aggregate in aqueous media which causes a high degree of ordered packing of water molecules around the hydrophobic compounds (i.e., a large positive Gibbs free energy driven by the adverse entropic effect on water). Thus, by associating with the hydrophobic compartments of circulating lipoproteins poorly soluble drugs can relieve the thermodynamic strain on the system and minimize the Gibbs free energy state.

Drugs may also associate with circulating lipoproteins through facilitated transport processes. This is mediated through by lipid transfer protein, often referred to as Cholesteryl Ester Transfer Protein (CETP). Other lipid transfer proteins have been reported for TG and phospholipid transport, however, CETP is the most studied and characterized transfer protein. CETP is a 74 kDa protein responsible for the facilitated transfer of neutral lipids (CE, TG) between lipoprotein classes [40,41]. The crystal structure of CETP was recently, published, providing significant insight into the mechanism of lipid transfer [42]. The protein appears to form a tunnel extending between adjacent donor and recipient lipoprotein particles to facilitate the molecular transfer of lipids. The 60 Å long hydrophobic tunnel has the capacity to concurrently ferry two cholesteryl ester molecules through its core [42]. The size and hydrophobicity of the tunnel suggest that indiscriminate neutral lipid binding could be possible and provides supporting evidence for the potential role of CETP in drug transport. Several lines of evidence have demonstrated the role of CETP in the transfer of amphotericin B, halofantrine and cyclosporine A between lipoprotein classes [43]. Similar processes are also anticipated to facilitate the transfer of poorly soluble anticancer drugs.

Alterations in plasma lipoprotein content and composition can also influence the extent that drugs associate with lipoproteins. Dyslipidemias (disruption in the normal distribution of lipid classes within plasma) can arise from disturbances caused by disease or medication. High levels of plasma LDL and VLDL will induce hypercholesterolemia and/or hypertriglyceridemia. In these conditions, LDL/VLDL can serve as a large depot for poorly soluble drugs. Conversely, in conditions where plasma lipoproteins are lowered, the plasma levels of poorly soluble drugs can be significantly reduced. The specific pharmacodynamic/pharmacokinetic perturbation drugs experience with dyslipidemia is dependent on multiple factors such as: hydrophobicity of the drug, the etiology of the dyslipidemia, tissue lipoprotein receptor expression, etc. As such, the therapeutic consequence of altered drug metabolism can be highly variable during dyslipidemia.

A few studies have also described lipoprotein lipid composition as an important factor influencing drug association. Increases in the TG to total cholesterol ratio was first found to increase drug (cyclosporine) association solely in the VLDL fraction [44]. Later investigations found that increases in this lipid ratio also increased drug association in the HDL pools [45]. These findings suggest that TGs may better solubilize drugs in the lipoprotein core than cholesterol moieties. Further studies are needed to validate these observations.

Drug association can occur within the aploar core or the external, more polar compartment of the lipoprotein particle. The relative preference of a drug to move into the apolar core or polar surface is determined by the logP (octanol/water partition coefficient) of the drug. Thus, hydrophobic drugs (high logP value) readily partition into the lipoprotein core [46,47]. This is a favorable compartment for drug transport as (i) it is ‘shielded’ from the external environment until receptor recognition/cell uptake and (ii) the core carrying capacity is considerable. Based on endogenous core lipid molecules, VLDL can carry an estimated 15,000 molecules of cholesterol esters and TG, LDL 1500 molecules, and HDL2 (10 nm diameter) 109 molecules [48]. On the more polar exterior amphiphilic drugs will associate with the phospholipid surface layer. Hydrophilic segments of the drug will orient near the phospholipid head groups, while the hydrophobic portions of the drug will associate with ‘buried’ fatty acyl chains of the phospholipids [46]. The surface characteristics of the phospholipid layer can also influence the partitioning of amphiphile drugs in the lipoprotein membrane monolayer. The surface layer of lipoproteins (VLDL and LDL) coexist in a liquid-order phase (rich in sphingomyelin and cholesterol) and a liquid-disorder phase (rich in glycerophospholipids) [49]. The more fluid and dynamic state of the liquid-disordered phase is more conducive for drug association/incorporation than the former liquid-order phase [50]. The polar surfaces of lipoproteins also have associated amphiphilic proteins (apolipoproteins) that differ in characteristics and surface coverage (Table 2). Conceivably, a drug could bind to a specific site on the apolipoprotein, analogous to albumin-drug binding. However, no specific examples of this type of interaction have been observed to date.

The natural drug interactions and the compartmentalized organization of lipoproteins strongly support the strategy of utilizing lipoproteins as drug delivery vehicles. As such, the next logical step would be to actively preload or formulate lipoproteins to carry exogenous agents for cancer treatment or detection. Several excellent papers have been written describing the formulation of lipoproteins with therapeutic or diagnostic agents [51,52,53,54,55,56].

## 5. Epidemiological Evidence for Lipoprotein and Cancer Relationship

There is a long history of investigations in human subjects examining the association of cancer and serum cholesterol levels (the majority of which is transported in circulating lipoproteins). A representative list of studies in the field has been presented as Table 3. In the following section we will explore the epidemiological data from some of the major studies to further mine the dynamic association between cholesterol and cancer.

## 6. Cholesterol and Cancer Risk

The largest study to date comprised of a cohort of over 1.2 million participants who enrolled in the Korean National Health Insurance Corporation medical evaluation between 1992–1995 and underwent biennial routine medical exams [56]. The study population which consisted of 53,944 men and 24,475 women were later diagnosed with cancer within the median follow-up time of 12.7 years. The data on total serum cholesterol (TSC) was stratified as high (>240 mg/dL) or low (<160 mg/dL) and adjusted for factors like lifestyle, habits, and fitness levels and comparisons between the groups were made as it related to cancer incidence. The data showed that across all cancer types, incidence of disease had a negative correlation with total cholesterol levels when high vs. low cholesterol groups were compared across both sexes (males: HR = 0.84; 95% CI = 0.81–0.86; females: HR = 0.91; 95% CI = 0.8–0.95). The study did a second inference excluding the patients who reported cancer within the first 5 years of study to exclude cases that might already have underlying cancers. Excluding patients from the first 5 years minimized the chances of including cases of cancer undiagnosed at the start of the study. The results of the analysis showed that the incidence was not affected much (males: HR = 0.87; 95% CI = 0.84–0.91; females: HR, 0.94; 95% CI = 0.89–1.00).

When the group looked at specific cancers, high TSC seemed to decrease the risk of liver cancer (males: HR = 0.42; 95% CI = 0.38–0.45; females: HR = 0.32; 95%CI = 0.27–0.39). However, since chronic liver disease can cause alterations in cholesterol metabolism, additional adjustments for liver health were made (ALT, AST hepatitis B surface antigen). The adjustments in assessments slightly attenuated the incidence (males: HR = 0.60; 95% CI = 0.54–0.67; females: HR = 0.46; 95% CI = 0.24 to 0.87), but did not alter the association with cholesterol dramatically. The incidence of stomach cancer (males HR = 0.87; 95% CI = 0.82–0.93; females: HR = 0.86; 95% CI = 0.77–0.97) also seem to be slightly significant across both sexes. On the other hand, the report suggests a positive association with breast cancer (HR = 1.17; 95% CI = 1.03–1.33) incidence in women and colon (HR = 1.24; 95% CI = 1.07–1.44) and prostate cancer (HR = 1.12; 95% CI = 1.03–1.33) in men. Excluding the early cases resulted a stronger correlation for breast cancer (HR = 1.21; 95% CI = 1.04–1.41) and colon cancers (HR = 1.28; 95% CI = 1.06–1.56) in females.

The overall conclusion of this study indicates that generally across different cancers there was an inverse relationship between cancer and cholesterol. However, the authors do mention that the overall results were heavily skewed by the number of liver cancer cases compared to other cancer types. For site specific cancers, liver and stomach cancers showed negative correlation. While breast cancers in females showed a positive correlation along with colon and prostate cancer in men. The significant power of this study comes from the large size of participants and the range of cholesterol values. The study also collected data and accounted for mitigating factor such as lifestyle, health status along with presence of liver disease. This study also had a 14 year follow up period which gave enough room for exclusion of early onset cases to eliminate undiagnosed cancer cases at the start of the study making this study a true risk analysis in contrast to correlative studies.

In another large study consisting of a European cohort of 577,330 individuals who were followed between 1972–2005 [57]. In this population study, 38,978 individuals reported having cancer in the mean follow-up time of 11.7 years [57]. The results showed that overall, cancer incidence was slightly inverse correlated with TSC (males; HR = 0.94; 95% CI = 0.88–1, females HR = 0.86; 95% CI = 0.79–0.93). Among the site specific data presented for males liver/bile duct (HR = 0.14; 95% CI = 0.07–0.29), pancreatic (HR = 0.52; 95% CI = 0.33–0.81), non-melanoma of skin (HR = 0.67; 95% CI = 0.46–0.95) and lymphatic/hematopoietic (HR = 0.68; 95% CI = 0.54–0.87) cancer showed significant inverse correlation. Similarly, in females, gallbladder (HR = 0.23; 95% CI: 0.08, 0.62), breast (HR = 0.70; 95% CI: 0.61, 0.81), skin melanoma (HR = 0.61; 95% CI: 0.42, 0.88), and lymphatic/hematopoietic (HR = 0.61; 95% CI: 0.44, 0.83) also showed inverse correlation.

A study in the state of California between 1964–1972 consisted of a large participant pool of 160,000 men and women spanning several cancer types [58]. This study showed that maximum risk was associated with high cholesterol in males with lymphoma (HR = 1.72; 95% CI 1.00–2.83) and females with cervical cancer (HR = 1.30; 95% CI 1.03–1.62) when lowest quintiles were compared to the higher ones. Among other cancer groups, prostate, lung, and pancreas in males and melanoma, ovarian, and lung cancers in females show modest increased risk. In contrast, risk marginally decreased for colon, rectum, melanoma and bladder in males and uterine cancers and lymphomas among females. Breast cancer showed no association in the data. Overall this study suggested a slightly higher risk of cancer associated across sexes (males HR = 1.03; 95% CI 0.97–1.10; females HR = 1.16; 95% CI 1.04–1.29)

**Figure 4:**
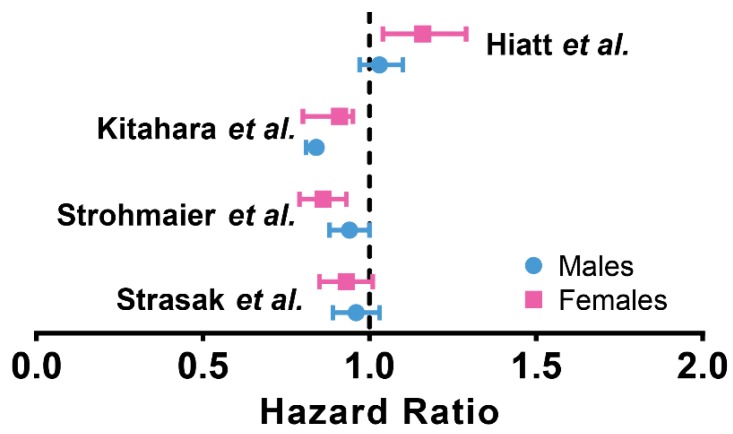
Hazard Ratios of Cholesterol-Cancer Association in Large Cohort Studies. Summary of hazard ratios in various large studies relating cancer and cholesterol. Note that majority of hazard ratio values are close to 1.

Overall, we see the majority of papers reporting an overall inverse correlation between cholesterol level and cancer incidence (Table 3). However, the hazard ratio for this correlation can be only characterized as marginal at best (Figure 4). Depending on the study, multiple site-specific cancers showed negative correlations. However, these relationships can also be classified as minor, apart from a few studies that report significant hazard ratios for liver cancer. In general, the hazard ratio associated with TSC levels were highly variable and hence provide minimal clinical predictive value for cancer.

In a sub-analysis of the European study consisting of 172,210 Austrian participants that were studied from 1985–2003 to determine the relationship between cholesterol and cancer risk in short term (<5 months) and long term (>5 months) [59]. In short term analysis, Strasak et al. observed a striking inverse correlation for overall cancer with compelling hazard ratios (males HR = 0.58; 95% CI = 0.43–0.78); females HR = 0.69; 95% CI = 0.49–0.99). Conversely, after 5 months, they reported low levels of risk similar to the previously discussed studies. For males the HR was 0.96 (95% CI 0.89–1.03) and for females HR was 0.93 (95% CI 0.85–1.01). This pronounced difference in hazard ratio for the short-term cohort can be attributed to “the preclinical effect” of cancer. The “preclinical effect” of cancer was proposed by McMichael et al. who stated that in “metabolic consequences of preexisting, undetected, cancer may be the cause of low serum cholesterol in those individuals at the time of their entry into the study” [60]. Thus, for true cancer-cholesterol risk studies, the hazard ratio can be skewed by patients who have undiagnosed cancer at the start of the study. This phenomenon can be seen in many of the cholesterol-cancer risk assessment studies. Additional studies by Hiatt et al. showed a strong pre-clinical effect among American patients who were diagnosed with cancer within two years of the study [58]. The overall relative risk for males increased approximately 2-fold among patients with the lowest cholesterol levels. Males with prostate, lung, and colon cancer showed the most striking increases in relative risk (3-fold, 2-fold, and 2-fold, respectively). Females with the lowest cholesterol levels also showed a slight increase in overall relative risk. Patients with carcinoma of the lung, colon, and uterine cancers displayed the highest levels of risk (3-fold, 2-fold, and 2-fold, respectively). Interestingly, when exclusion periods were extended out to 5 years, as in the Korean and the European study, the pre-clinical effect was not observed.

While the risk to develop cancer from cholesterol is modest at best, after the onset of cancer, cholesterol levels are profoundly affected.

## 7. Cholesterol Levels During Cancer

**Figure 5:**
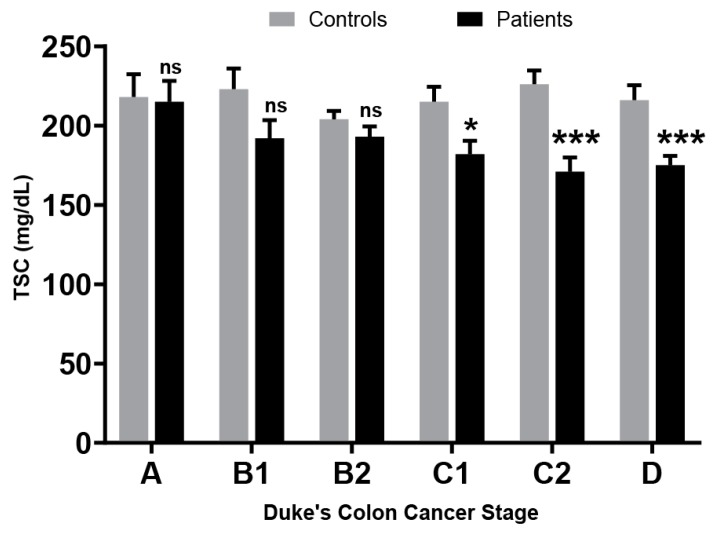
Total serum cholesterol levels observed by Miller et al. in colon cancer patients vs. controls. Error bars denote Standard Deviation. *p* values for paired t-test * = < 0.05, *** = < 0.001. ns = not significant.

There are several site-specific cancer studies showing relationships of cholesterol levels with different stages of cancer progression. Dessi et al. [70], while studying several hematologic malignancies, found a decrease in HDL-C in patient vs. control samples. They also report a negative correlation between cell proliferation based on clinical severity of the disease and serum HDL-C. Among solid tumors, hypocholesteremia was also seen by Umeki et al. in non-resectable lung cancer patients [75]. In addition, Dessi et al. [74], showed increased cholesterol in surgically removed tumoral tissue and a concomitant lower serum HDL-C in the lung cancer cohort. The study also found a 2-fold increase in free cholesterol and 3.5-fold increase in esterified cholesterol in the tumor compared to normal tissues. The esterified form of cholesterol is the primary means of cholesterol storage in rapidly dividing cells to provide an immediate reservoir of cholesterol for new cell membranes [74]. These two studies collectively show that tumors actively accumulate cholesterol leading to lower TSC. Miller and colleagues studied TSC and HDL-C in colon cancer as it progresses from stage A to D (Duke’s Staging system) and saw decreasing levels as the disease advanced (Figure 5) [61]. This overall relationship between cholesterol and cancer was dissected further by Kokoglu et al. in the setting of breast cancer. They showed lower cholesterol levels in Stage I patients (187.3 mg/dL vs. 201.4 mg/dL in healthy controls) and showed further depletion of TSC in Stage IV (159.7 mg/dL) [66]. Similar stage related decrease in TSC was also seen in cervical cancer patients [77]. Potischman et al. observed that patients at Stages I and II had a mean TSC level of 160 mg/dL (95% CI = 154–166 mg/dL) while those in stage III and IV had TSC mean of 151 mg/dL (95% CI = 144–157 mg/dL) and 148 mg/dL (95% CI = 130–167 mg/dL), respectively. This negative correlation between cancer progression and cholesterol is most striking when we study the relationship during metastasis or stage IV. In a study by Kritchevsky et al. [72], patients with distant metastasis had lower TSC and LDL-C than patients with more localized disease. These observations can be explained by the fact that as cancer progress towards metastasis in stage III and Stage IV, the proliferative activity and tumor burden increase further exasperating the demand for cholesterol. Cancer cells meet this demand by increasing cholesterol uptake from their environment through upregulating lipoprotein receptors like LDLR. Accompanying the higher LDLR activity in tumor tissues, both Vitols [62] and Peterson [63] reported lower TSC in hematological and solid tumors respectively. These findings support the notion that cancer induced hypocholesteremia may be the result of tumor sequestering of plasma cholesterol.

## 8. Cholesterol Levels Following Cancer Treatment

Given that established cancers induce hypocholesteremia, effective anti-cancer treatments should reverse this lipid disturbance. Indeed, Vitols et al. reported that plasma levels of cholesterol increased as patients responded to chemotherapy that diminished the count of leukemic cells [62]. Additionally, a case study in the above-mentioned paper reported that when leukemic cells were removed from a patient by leukapheresis, the LDL-C was normalized. Alexopoulos et al. also reported increases in TSC and LDL-C after patients with varying types of cancer went through chemotherapy [73]. The overall change in TSC across all cancer types increased from 190 ± 45 mg/dL pre-treatment to 215 ± 50 mg/dL post treatment. The normalized TSC levels were maintained in patients throughout remission. Similar results were also reported in several other studies in hematologic and solid tumors [78,82,92].

In summary, these studies show a direct association and causality between tumor burden and the observed alterations in circulating cholesterol.

## 9. Molecular Role of Cholesterol in Cancer

Cholesterol is an essential building block for the construction of new cell membranes, and thus is necessary for rapidly proliferating cells. Cancer cells have altered composition and lipid metabolism when compared to normal cells owing to upregulated lipid and cholesterol biosynthesis [95,96]. One group has suggested that high cholesterol content in the lipid raft component of cancer cells poses a unique vulnerability for cancer cells [97]. This observation has led to the idea that certain drugs that perturb cholesterol homeostasis might be employed as potential treatments for cancer [98,99]. The therapeutic utility of inhibiting cholesterol biosynthesis or access is supported by the findings that oncogenic pathways drive cancer cells to accumulate more cholesterol. In contrast, there are also findings that cholesterol itself serves as signaling molecule to induce cancer aggressiveness. The following sections will examine these two seemingly contrasting observations.

## 10. Cholesterol as an Oncogenic Driver

In this section we will examine the evidence of cholesterol and its derivatives as signaling molecules. One such example of this phenomenon was demonstrated by Huang et al. when they showed that cholesterol directly stimulates Smoothened (SMO) leading to activation of Hedgehog (SHH) signaling. Smoothened is transmembrane G-protein coupled receptor protein that is normally repressed by the tumor suppressor Patched (*PTCH1*). When Hedgehog signaling is activated, Smoothened transduces signaling, leading to activation of GLI transcription factors that stimulate cancer cell growth [100]. Huang et al. further showed that Smoothened is activated by cholesterol and various cholesterol derivatives, such as oxysterols, which are naturally occurring oxidized forms of cholesterol [101]. While many of these molecules have cellular concentrations below the EC_50_ dose to activate Smoothened; in certain cases, such as cancer, cholesterol itself may be able to activate Smoothened. It remains unclear whether cholesterol mediated activation of Hedgehog signaling is enough to drive tumorigenesis, or if it simply facilitates tumor progression.

Cholesterol also plays a role in activating other G-protein coupled receptors (GPCRs). Guixà-González et al. showed that cholesterol binds directly to the Adenosine A_2A_ GPCR [102]. This group further suggested that cholesterol plays a role in allosteric regulation of GPCRs and could potentially even activate signaling. In a recent paper by Moon et al. cholesterol signaling through GPCRs was implicated in androgen independent metastasis in prostate cancer [103].

It is well documented that cholesterol and its derivatives can activate both the Estrogen Receptor (ER) and Estrogen Related Receptors (ERRs) [104,105]. The Estrogen Receptor is known as a promiscuous receptor and has many ligands, several of which are derived from cholesterol including its primary ligand estrogen [106]. Nelson et al. showed that a specific derivative of cholesterol, 27-hydroxycholestrol (27HC) serves as an ER ligand in the context of breast cancer [105]. This group further showed that 27HC is enough to drive the growth of MCF7 xenograft tumors. Nelson et al. then showed that the enzyme that makes 27HC (CYP27A1) is correlated with higher tumor grade and metastasis. While Nelson et al. suggested that inhibiting synthesis of 27HC may prove to be a potential therapy for breast cancer, this molecule is probably not the primary driver in breast cancer, but is likely to be a method that contributes to resistance to therapy as suggested by Simigdala et al. [107].

Estrogen Related Receptors (ERRs) are a family of nuclear receptor transcription factors that are not as well studied in the context of cancer. The most well understood receptor in this family is ERRα; with ERRβ and ERRγ being less well studied [104]. One group, Wei et al. showed that one of many ligands for ERRα is cholesterol [108]. They observed that when cells are depleted of cholesterol, ERRα transcriptional activity disappears. Furthermore, they showed that statin treatment and resulting cholesterol depletion shut down ERRα transcription. ERRβ and ERRγ do not yet have an identified natural ligand, but do bind synthetic ligands such as 4-hydroxy-tamoxifen (4-OHT) and bisphenol-A [109,110]. Not much is known about the relationship between cholesterol and ERRβ or ERRγ, but due to sequence homology with ERRα it is likely that cholesterol or its derivatives have some ability to activate these two receptors.

**Figure 6:**
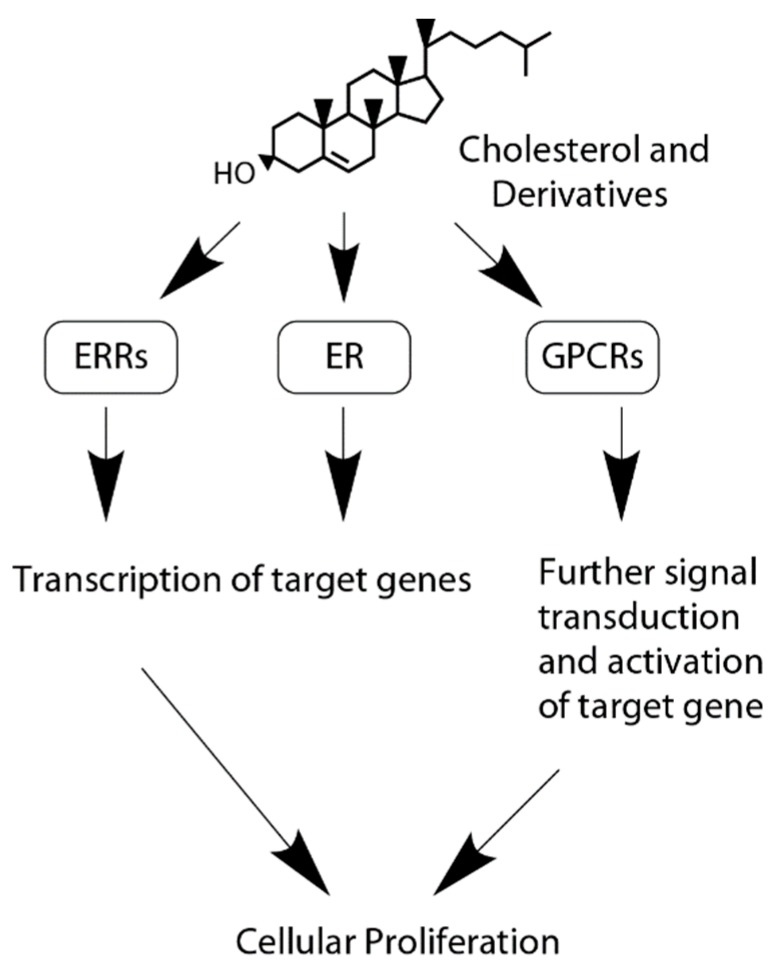
Signaling functions of cholesterol and cholesterol derivatives. Cholesterol and its derivatives interact with estrogen related receptors (ERRs), the estrogen receptor (ER), and G-protein coupled receptors (GPCRS) to induce more oncogenic signaling mediated by transcriptional activation of further downstream signaling.

In summary, cholesterol can activate three different signaling pathways (GPCRs, ER, and ERRs) in cancer as shown in Figure 6.

Cholesterol also forms a major component of lipid rafts, which is central to many processes in signal transduction [111]. Lipid rafts are generally small ranging in size from 10–200 nm, and are densely packed with cholesterol, proteins, and sphingolipids [112]. Lipid rafts are generally represented as floating collections of various signaling proteins that can be transported to and from the cell membrane to facilitate signaling [113]. Many canonical drivers of cell proliferation have signaling that has been localized to lipid rafts including Epidermal Growth Factor Receptor (EGFR), Insulin-like growth factor receptor (IGFR), Hedgehog, and H-RAS [113,114,115]. AKT which functions downstream of various receptors in cancer, also has been localized to lipid rafts, and it has been shown that AKT signaling stops when cellular cholesterol is depleted [99]. Concurrently, it has also been shown that HER2 signaling is localized to cholesterol rich domains in cell membranes, suggesting that cholesterol plays a role is helping facilitate HER2 dependent oncogenic signaling [95]. Thus, lipid rafts may present a good target to treat cancer cell as suggested by Li et al. [97].

Another potential mechanism whereby cholesterol and lipids can promote carcinogenesis through lipid peroxidation. Oxygen mediated breakdown of unsaturated lipids is known to create many reactive and potentially mutagenic substances. Tseng et al. showed that a high cholesterol diet can lead to induction of lipid peroxidation that can help contribute to carcinogenesis [116]. While the study did not go into the exact mechanism of how lipid peroxidation induces cancer, the majority of lipids and cholesterol derivatives have been shown to undergo lipid peroxidation [117]. One cholesterol derivative 7-dihydro-cholesterol is particularly vulnerable to auto-oxidation, and likely plays a part in cholesterol mediated mutagenesis. More recent studies have proposed that oxidized low-density lipoprotein may be to blame [118]. One such example by Khaidakov and Mehta showed that stimulation of mammary epithelial cells with oxidized LDL increased expression of miR21, which then inhibits function of PTEN resulting in activation of AKT [119].

## 11. Cholesterol Accumulation Driven by Oncogenic Signaling

**Figure 7:**
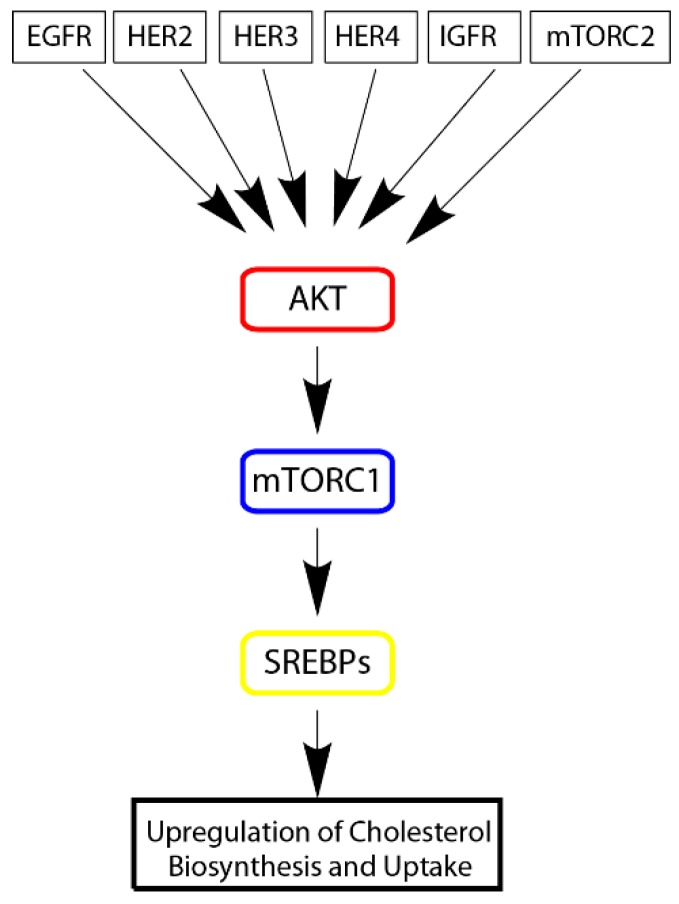
AKT, Master Regulator of Cholesterol Accumulation. AKT plays a central role in receiving signals from various oncogenic drivers (Epidermal Growth Factor Receptor (EGFR), HER2, HER3, HER4, Insulin-like growth factor receptor (IGFR), and mTORC2) and then activating Molecular Target of Rapamycin Complex 1 (mTORC1) which then leads to activation of Sterol Regulatory Binding Protein (SREBPs) that then upregulate cholesterol synthesis and uptake.

Cholesterol homeostasis in cells is regulated by SREBP1 and SREBP2 proteins belong to the Sterol Regulatory Binding Protein family (SREBPs), which are transcription factors that respond to intracellular levels of cholesterol [120]. SREBP1 was shown to regulate levels of LDLR in cells as do other proteins of the SREBP family who perform similar functions [121]. Subsequent observation by Porstmann et al. found that that SREBPs are regulated by the Molecular Target of Rapamycin Complex 1 (mTORC1) as a result of AKT oncogenic signaling [122]. This finding suggests that mTORC1 serves as a central regulator of cancer cell metabolism including cholesterol import and biosynthesis. mTOR signaling is overactive in many cancers, including breast, prostate, lung, liver, and kidney cancers. As such, this mechanism of upregulating cholesterol biosynthesis is likely prevalent in these cancers [123]. Later studies also report this, Yue et al. reported that PTEN loss results in unabated PI3K/AKT signaling that induces the accumulation of cholesterol [124]. Yue et al. further reported that patient samples showing high cholesterol accumulation displayed a more aggressive phenotype. In another context, neuregulin activated ERBB4 (HER4) was shown to induce activity of SREBP-2 which resulted in higher low-density lipoprotein uptake [125]. Given that ERBB4 is one of many upstream regulators of AKT signaling, the cholesterol enriching tumor phenotype should be common in many ERBB4 expressing cancers [126]. In many cancers AKT seems to serve as a master regulator of cellular metabolism; including both of catabolic (cellular energetics) and anabolic processes, such as cholesterol biosynthesis (Figure 7). Interestingly, AKT signaling can be abated by using simvastatin which would suggest that cholesterol pays a role in allowing AKT signaling to proceed [99]. This could be explained simply by the fact that cholesterol forms a large component of lipid rafts and that AKT signaling is localized to lipid rafts [127]. While this may be the simple explanation, the relationship between cholesterol and AKT signaling is probably more complex, owing to the findings that cholesterol serves as a signaling molecule.

## 12. Cholesterol Feedback Loop, Both Sides of the Same Process

**Figure 8:**
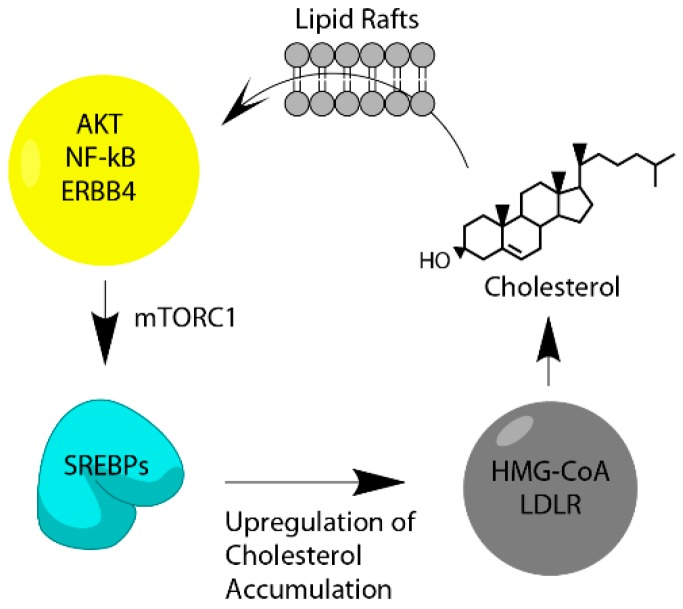
Cholesterol Feedback Loop. AKT signaling drives increases in cholesterol biosynthesis and uptake mediated by mTORC1 and SREBPs which leads to increased levels of cholesterol that activates more oncogenic signaling leading to a more aggressive tumor.

Within the cancer cell, cholesterol can initiate signaling functions through GPC transmembrane receptors, Hedgehog, ERRs, and ER. The idea of a cholesterol feedback loop was proposed by He and colleagues in 2017 in the context of hepatocellular carcinoma [128]. They proposed that inflammatory signaling mediated by NF-κB induces cholesterol accumulation by activating transcription of SREBP-2, thereafter cholesterol further activates NF-κB resulting in more pro-inflammatory signaling. Thus, once oncogenic signaling begins it starts cellular proliferation which enhances the need for more cholesterol. The acquired cholesterol is then able to drive the cell towards a more malignant phenotype (Figure 8.). A similar process seems to occur with AKT signaling owing to the finding that AKT signaling can be shut down by inhibiting cholesterol biosynthesis with a statin and by activation of the liver X receptor [99,129,130]. Furthermore, in HER2 enriched breast cancer, cholesterol seems to stabilize the HER2 receptor in the membrane suggesting another method that cholesterol can use to induce more oncogenic signaling [95]. Thus, this positive feedback loop seems to occur in multiple cancers, indicating that this may be a general molecular strategy of potentiating tumor growth and metastasis. It also follows that interventions that take advantage of a tumor’s need for cholesterol may be a useful therapeutic approach for eliminating cancer cells.

## 13. Conclusions

In addition to the delivery of natural lipids, lipoproteins may also be used to facilitate the systemic transport of drugs or diagnostic agents. This notion of a lipoprotein-mediated drug delivery system is supported by multiple lines of evidence in this review. Pharmacological studies have shown that thermodynamic forces naturally drive the interaction and association of circulating lipophilic and amphiphilic drugs with lipoproteins. Further supporting this association, is the compartmentalized organization of the lipoprotein readily facilitates the incorporation of exogenous molecules into its structure. Surprisingly enough, malignant cells avidly acquire plasma lipoproteins from the circulation. Clinical epidemiologic data among patients with diagnosed cancer strongly demonstrate the high sequestering of plasma cholesterol/lipoproteins by tumors. Furthermore, these clinical findings are corroborated by molecular data that substantiates that cholesterol uptake and accumulation in tumors is driven by the oncogenic driver AKT. Conversely, cholesterol has also been shown to serve as a key signaling molecule in tumor progression. Thus, in a reciprocal positive feedback loop cholesterol accumulation further drives tumor aggression. Collectively, these finding provide strong scientific reasoning for the adoption of lipoproteins as drug delivery vehicles for cancer treatment and detection.

## Figures and Tables

**Table 1:** Physiochemical Properties of Lipoproteins.

	Chylomicrons	VLDL	LDL	HDL
Density (g/mL)	<0.95	0.95–1.006	1.019–1.063	1.063–1.210
Diameter (nm)	>75	30–80	18–25	7–14
Protein	1–2	8–10	20–25	52–60
TG	80–95	45–65	4–8	2–7
Cholesterol	1–3	4–8	6–8	3–5
Phospholipid	3–6	15–20	18–24	26–32
Cholesteryl ester	2–4	6–10	45–50	15–20
Electrophoretic mobility	-	Pre-β	β	A

Physiochemical Properties of Lipoproteins. VLDL—very low density lipoprotein; LDL—low density lipoprotein; HDL—high density lipoprotein; TAG—triacylglycerol. Expressed in % dry weight.

**Table 2:** Properties of Major Human Apolipoproteins.

Apolipo-protein	Mw (kDa)	Plasma Conc (mg/dL)	Lipoprotein Distribution	Function (s)
ApoA1	29	130	All HDL subclasses	cholesterol efflux; LCAT activation
ApoA2	17.4	40	HDL-1, HDL-2, HDL-3	Inhibition of apoA1 activity
ApoA4	44.5	15	Chylomicrons	LCAT activation
ApoB48	241	Transient	Chylomicrons	Chylomicron secretion
ApoB100	512	80–250	VLDL, LDL	VLDL secretion; LDL receptor ligand
ApoC1	6.6	3-6	HDL, LDL	LCAT activation
ApoC2	9	3–12	VLDL, HDLs	Activation of LPL
ApoC3	9	12	VLDL, HDLs	Inhibition of apoC2 activity, VLDL uptake
ApoD	19	10–12	HDL	Several Proposed
ApoE	34	5–7	VLDL, HDL-1	Cholesterol efflux; LDL receptor ligand

Properties of Major Human Apolipoproteins. VLDL—very low density lipoprotein; LDL—low density lipoprotein; HDL—high density lipoprotein; LCAT—lecithin–cholesterol acyltransferase; LPL—lipoprotein lipase.

**Table 3:** Major Studies Showing Cancer and Lipoprotein Correlations.

	Author	Year	Cancer Site	Major Conclusions
1	Miller, S. R., et al. [61]	1981	Colon	Colon cancer patients had TSC < Controls
2	Vitols, S., et al. [62]	1985	Blood	LDLR expression was high in leukemic cells. TSC levels back to normal after chemotherapy
3	Peterson, C., et al. [63]	1985	Blood	
4	Budd & Ginsberg [64]	1986	Blood	TSC, LDLC & HDLC lower in patients than controls. TSC, LDLC and HDLC back to normal during remission
5	Neugut, A. I., et al. [65]	1986	GI	TSC-Patient < Controls
6	Bani, I. A., et al. [66]	1986	Breast	TSC - Patient > Control. HDLC-Patient < Controls
7	Reverter, J. C., et al. [67]	1988	AML	LDLR expression was high in leukemic cells. TSC levels back to normal after chemotherapy
8	Marini, A., et al. [68]	1989	Blood	TSC-Patient < Controls
9	Rudling, M. J., et al. [69]	1990	Head	LDLR activity on tumor high
10	Dessi, S., et al. [70]	1991	Blood	HDLC patients < Controls. HDLC levels inversely correlated with cell proliferation.
11	Shokumbi, W. A., et al. [71]	1991	Blood (ALL)	HDLC patients < Controls
12	Kritchevsky, S. B., et al. [72]	1991	Multiple	TSC decreased in patients before diagnosis
13	Alexopoulos, C. G., et al. [73]	1992	Multiple	Positive response to chemotherapy correlated with increase in TSC
14	Dessi, S., et al. [74]	1992	Lung	Tumor had 2-fold cholesterol. HDLC patients < controls
15	Umeki, S. [75]	1993	Lung	TSC and HDLC patients < controls
16	Bayerdorffer, E., et al. [76]	1993	Colorectal	HDLC patients < controls; LDLC VLDLC patients > Controls
17	Potischman, N., et al. [77]	1994	Cervical	TSC in patients Stage I > Stage II > Stage IV
18	Baroni, S., et al. [78]	1994	Blood (ALL)	TSC HDLC patients < controls. Complete remission correlated with increase in TSC and HDLC
19	Kokoglu, E., et al. [79]	1994	Breast	TSC VLDLC patients < controls. HDLC LDLC Stage IV < Stage I patients. VLDL Stage IV > Stage I Patients
20	Juliusson, G., et al. [80]	1995	Blood (HCL)	TSC LDLC inversely correlated with tumor burden
21	Niendorf, A., et al. [81]	1995	Colon	TSC 12months post-surgery > 3 months post-surgery. Resected tumor had higher LDLR mRNA
22	Dessi, S., et al. [82]	1995	Multiple	HDLC patients < Controls. HDLC remission > diagnosis
23	AvallLundqvist, E. H. and C. O. Peterson [83]	1996	Ovarian	TSC at diagnosis < post-surgery < remission
24	Grieb, P., et al. [84]	1999	Brain	No reduction in TSC
25	Siemianowicz, K., et al. [85]	2000	Lung	TSC Patients < Controls
26	Siemianowicz, K., et al. [86]	2000	Lung	No difference in LDLC
27	Fiorenza, A. M., et al. [87]	2000	Multiple	TSC LDLC HDLC patients < Controls
28	Abiaka, C., et al. [88]	2001	Multiple	TSC patients < Controls
29	Caruso, M. G., et al. [89]	2001	Colorectal	LDLR protein and mRNA detected on tumor tissue. LDLR mRNA higher in tumors not expressing protein
30	Tomiki, Y., et al. [90]	2004	GI	TSC LDLC patients < Controls
31	Michalaki, V., et al. [91]	2005	Multiple	HDLC patients < Controls in Breast Cancer
32	Muntoni, S., et al. [92]	2009	Multiple	HDLC patients < Controls
33	Li, X., et al. [93]	2018	Breast	TSC HDLC LDLC patients < Controls
34	Carr, B. I., et al. [94]	2018	Liver	HDLC associated with Tumor Aggressiveness Index

Major studies showing cancer and lipoprotein correlations. Study citation shown with year of publication and cancer focused on by the study along with the major conclusion by the study.

## Abbreviations

27HCL	27-Hydroxycholestrol
4-OHT	4-Hydroytamoxifen
AKT	Rac-Alpha Serine/Threonine-Protein Kinase
ALL	Acute Lymphocytic Leukemia
AML	Acute Myeloid Leukemia
BDDCS	Biopharmaceutics Drug Disposition Classification System
CE	Cholesterol Ester
CETP	Cholesteryl Ester Transfer Protein
CI	Confidence Interval
DHA	Docosahexaenoic Acid
EGFR	Epidermal Growth Factor Receptor
ER	Estrogen Receptor
ERBB4/HER4	Epidermal Growth Factor Receptor Family Member 4
ERRS	Estrogen Related Receptors
GLIs	glioma-associated oncogene
GPCRS	G-Protein Coupled Receptor
GTPase	Guanosine Triphosphate Hydrolase Enzyme
HDL	High Density Lipoprotein
HDL-C	High Density Lipoprotein Cholesterol
HER2	Epidermal Growth Factor Receptor Family Member 2
HR	Hazard Ratio
IGFR	Insulin Growth Factor Receptor
IQGAP1	Ras Gtpase-Activating-Like Protein 1
LCAT	Lecithin–Cholesterol Acyltransferase
LDL	Low Density Lipoprotein
LDL-C	Low Density Lipoprotein Cholesterol
LDLR	Low-Density Lipoprotein Receptor
LOX-1	Lectin-Type Oxidized Low Density Lipoprotein Receptor 1
LPL	Lipoprotein Lipase
mg/dL	Milligram Per Deciliter
mTORC1/2	Molecular Target of Rapamicin Complex 1 and 2
PI3K	Phosphoinositide 3-Kinase
PTCH1	Patched
PTEN	Phosphatase And Tensin Homolog
SHH	Hedgehog
SMO	Smoothened
SREBPs	Sterol Regulatory Binding Protein Family
TG	Triglycerides
TSC	Total Serum Cholesterol
VLDL	Very Low Density Lipoprotein Cholesterol
